# Biomacromolecular Profile in Human Primary Retinal Pigment Epithelial Cells—A Study of Oxidative Stress and Autophagy by Synchrotron-Based FTIR Microspectroscopy

**DOI:** 10.3390/biomedicines11020300

**Published:** 2023-01-21

**Authors:** Natasha Josifovska, Sofija Andjelic, Lyubomyr Lytvynchuk, Xhevat Lumi, Tanja Dučić, Goran Petrovski

**Affiliations:** 1 Center for Eye Research and Innovative Diagnostics, Department of Ophthalmology, Oslo University Hospital, and Institute for Clinical Medicine, Faculty of Medicine, University of Oslo, 0450 Oslo, Norway; 2 Eye Hospital, University Medical Center, 1000 Ljubljana, Slovenia; 3 Department of Ophthalmology, Justus Liebig University, University Hospital Giessen and Marburg GmbH, 35390 Giessen, Germany; 4 Karl Landsteiner Institute for Retinal Research and Imaging, 1030 Vienna, Austria; 5 CELLS-ALBA, Carrer de la Llum 2-26, Cerdanyola del Valles, 08290 Barcelona, Spain; 6 Department of Ophthalmology, University of Split School of Medicine and University Hospital Centre, 21000 Split, Croatia

**Keywords:** synchrotron-based FTIR microspectroscopy, age-related macular degeneration, human primary RPEs, autophagy, oxidative stress

## Abstract

Synchrotron radiation-based Fourier Transform Infrared (SR-FTIR) microspectroscopy is a non-destructive and chemically sensitive technique for the rapid detection of changes in the different components of the cell’s biomacromolecular profile. Reactive oxygen species and oxidative stress may cause damage to the DNA, RNA, and proteins in the retinal pigment epithelium (RPE), which can further lead to age-related macular degeneration (AMD) and visual loss in the elderly. In this study, human primary RPEs (hRPEs) were used to study AMD pathogenesis by using an established in vitro cellular model of the disease. Autophagy—a mechanism of intracellular degradation, which is altered during AMD, was studied in the hRPEs by using the autophagy inducer rapamycin and treated with the autophagy inhibitor bafilomycin A1. In addition, oxidative stress was induced by the hydrogen peroxide (H_2_O_2_) treatment of hRPEs. By using SR-FTIR microspectroscopy and multivariate analyses, the changes in the phosphate groups of nucleic acids, Amide I and II of the proteins, the carbonyl groups, and the lipid status in the hRPEs showed a significantly different pattern under oxidative stress/autophagy induction and inhibition. This biomolecular fingerprint can be evaluated in future drug discovery studies affecting autophagy and oxidative stress in AMD.

## 1. Introduction

Age-related macular degeneration (AMD) is one of the leading causes of vision loss and blindness around the globe. Aging is the most important risk factor for the development and progression of the disease. However, there are also other important factors as inflammation, oxidative stress, and genetic predisposition, which have been linked to the disease [1]. AMD is characterized by changes in the choroid and the retinal outer layers, particularly the RPE and the photoreceptor layer. Its exact mechanism is still not entirely clear, however, apoptosis, autophagy, and necroptosis have been claimed to play a role in AMD pathogenesis. Autophagy is related to apoptosis and necroptosis, as well as AMD, while the interplay of the different pathological mechanisms of the disease needs further clarification [2]. It is a protective mechanism important for cellular homeostasis mostly activated under conditions of stress, inflammation, proteasomal inhibition, and starvation [3,4,5,6,7]. As a self-digestive mechanism, autophagy is important for the clearance of cellular debris, and in that way, maintenance of cellular vitality and tissue homeostasis. Under the conditions of oxidative stress, autophagy is involved in cellular turnover and activation of the proteins responsible for counteracting it, such as the NFE2L2, which can interact with an important autophagic protein, p62 [1,2,8]. The RPEs are burdened by an increased accumulation of debris throughout aging. By digesting photoreceptor outer segments (POS), the RPEs maintain the structural and functional integrity of the retina. The blockage of debris clearance by the autophagolysosomal pathway has been of interest as one of the most important mechanisms in AMD development, and considering its potential in disease treatment [2]. Abnormalities in the RPE and Bruch’s membrane in the early stages of the disease, are usually followed by further degeneration as the disease progresses [9]. A decline in the autophagic flow through age can lead to the accumulation of damaged proteins and mitochondria in the RPE cells, which results in their dysfunction [10]. As a result of high oxygen consumption, light exposure, and high mitochondrial activity the RPE cells throughout their lifecycle are subjected to oxidative stress, and the release of reactive oxygen species (ROS) [5]. Since the expression of anti-oxidative enzymes and other antioxidant production in RPEs with aging decreases, this can cause significant oxidative damage resulting in cell death and AMD development [11,12,13]. One of the major naturally produced ROS in the human eyes, which level increases under pathological conditions is hydrogen peroxide (H_2_O_2_) [14]. Increasing evidence for its relation with high autophagic activity represents a well-known in vitro model for triggering oxidative stress [3,15,16]. Besides oxidative stress, autophagy can be triggered by environmental changes (e.g., nutrient deprivation, influence of hormones). A convenient inducer of autophagy for experimental purposes is rapamycin (Rap), which is a lipophilic macrolide antibiotic [17]. Inhibition of the proteasomal- and the autophago-lysosomal- fusion can be carried out also by bafilomycin A1 (Baf) or chloroquine [18,19], respectively.

In this study, we evaluate the complete bio-macromolecular information at a cellular level, i.e., proteins, lipids, and nucleic acids composition and their conformational changes in untreated primary human RPEs (hRPEs) compared to cells in which autophagy was induced or inhibited as a study model for AMD. To determine the molecular and cellular changes in hRPEs under such treatments, synchrotron radiation-based Fourier Transform Infrared (SR-FTIR) microspectroscopy was used, which is a well-known high-throughput non-destructive technique for detecting changes in the functional groups of biomolecules belonging to the cell or tissue components in situ. The differential fingerprint of the functional groups of lipids (2800–3050 cm^−1^), proteins and esters (1485–1760 cm^−1^), and nucleic acids and carbohydrates (890–1310 cm^−1^) allowed characterization of the biomolecular pattern in our studied samples [20]. By checking the changes in the biomacromolecular profile of hRPEs, one can discover how novel therapies that target and impact oxidative stress and autophagy in AMD can affect the molecular fingerprint in the hRPEs, and possibly detect changes from early/dry form to late/wet form of AMD.

## 2. Materials and Methods

### 2.1. Samples

The cells used in this study were hRPE cells isolated from cadaveric eyes and cultured in F-12 DMEM culture media (Gibco, Life Technologies Limited, Inchinnan, UK) supplemented with 10% FBS and 1% Antibiotic/antimycotic (Sigma-Aldrich, St. Louis, Missouri, USA) at 37 °C in a saturated humid atmosphere with 5% CO_2_. This study was performed in accordance with the Guidelines of the Declaration of Helsinki. The study protocol regarding the isolation, cultivation, and use of hRPE cells was approved by the Regional Committees for Medical and Health Research Ethics for the Center for Eye Research, Department of Ophthalmology, Oslo University Hospital and University of Oslo (Oslo, Norway) with Ref. nr.: 2017/418.

Three donors with passage number 0 were included in the experiment. The cells underwent different treatment conditions before being prepared for SR-FTIR studies.

100,000 cells were seeded on each 10 × 0.5 mm Calcium Fluoride (CaF_2_) slide (Crystran Ltd., Poole, Dorset, UK) for the following study groups: control (untreated), treatment with H_2_O_2_ (Sigma-Aldrich, St. Louis, Missouri, USA), Rap (Santa Cruz Biotechnology, Heidelberg, Germany), Baf (Sigma-Aldrich, St. Louis, Missouri, USA), and Baf+H_2_O_2_.

The model of induction of oxidative stress was made by treating hRPEs with 0.6 mM H_2_O_2_ for 30 min. Autophagy was induced by treating the cells with 50 nM Rap for 24 h, while autophagy inhibition was achieved by using 50 nM Baf for 24 h, as well as Baf + 0.6 mM H_2_O_2_ treatment for 30 min followed by Baf for an additional 23 h 30 min. At the end of the treatments, supernatants were discarded and the cells were fixed in 4% paraformaldehyde-PBS solution (Biotium, Fremont, California, USA) for 20 min. Furthermore, the cells were washed 2 times with Milli-Q water, dried under sterile conditions in the laminar flow at room temperature, and stored over silica gel prior to the measurements at the ALBA synchrotron.

### 2.2. SR-FTIR Microspectroscopy

FTIR measurements were performed at the MIRAS beamline of the ALBA Synchrotron, Barcelona, Spain. A Hyperion 3000 coupled to a Vertex 70 spectrometer (Bruker, Ettlingen, Germany) equipped with a Mercury Cadmium Telluride (MCT) detector and with an aperture size of 10 × 10 µm^2^ was used for the spectroscopic data collection. The synchrotron infrared light was focused on the samples by using a 36× Schwarzschild objective coupled to a 36× Schwarzschild condenser (NA = 0.52). In total 35–37 individual cells for each study group were selected using the microscope and each spectrum was acquired after co-adding 252 scans at a spectral resolution of 4 cm^−1^. Before every 10 scans, a background was taken from a cell-free area.

The spectroscopic maps were made with an aperture size of 8 × 8 µm^2^ and step size of 4 × 4 µm^2^ over the single cells, and specific integrated regions are shown as assigned later in the figures: the ratio of CH_2_ and CH_3_ asymmetric vibration bands and carbonyl (C=O groups).

We used the OPUS 8.2 (Bruker, Ettlingen, Germany) software package for data collection and analysis of cell maps. The Quasar software package Version 1.2.0 (Bioinformatics Laboratory of the University of Ljubljana, Slovenia) was used for the statistical evaluation and spectra preprocessing. The data was pre-processed by Rubber-band background correction and the vector normalized spectra for each group of bio-macromolecules: lipids (2800–3050 cm^−1^), proteins and ester groups (1485–1760 cm^−1^), and fingerprint which includes nucleic acids and carbohydrates (890–1310 cm^−1^). The second derivative of spectra was calculated by using the Savitzky-Golay algorithm (window 11, third polynomial order, derivative order 2).

## 3. Results

The analyzed infrared spectra consisted of several bands arising from the vibration of different molecular groups belonging to proteins, nucleic acids, carbohydrates, and lipids, indicating that the cells had a uniquely rich biochemical composition.

**Figure 1:**
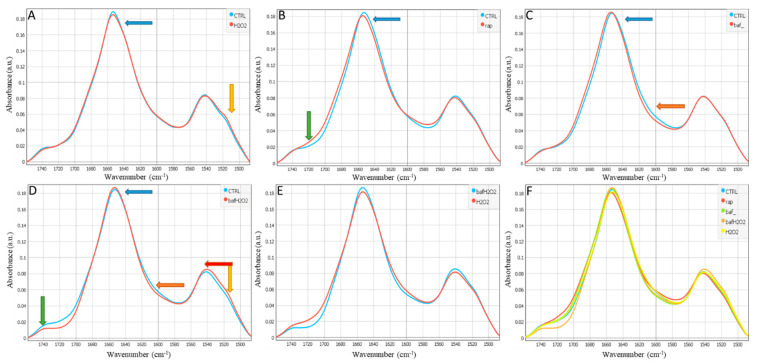
Average spectra of the protein and ester region (1485–1760 cm^−1^) in hRPE cells: (**A**) Control (untreated) vs. H_2_O_2_, (**B**) Control vs. Rap, (**C**) Control vs. Baf, (**D**) Control vs. Baf+H_2_O_2_, (**E**) Baf+H_2_O_2_ vs. H_2_O_2_, and (**F**) all conditions are being shown. Representative data of a number of cells per condition (N = 35–37 cells).

The hydrogen bonds between peptides are very important in the protein area for which degree of strength is represented by the position of the Amide I bands. A higher wavenumber corresponds to weaker hydrogen bonding and, therefore, less ordered protein structure. Changes in the Amide I position to a higher wavenumber can indicate protein disordering under experimental conditions [21]. Figure 1 and Table S1, the latter adapted from Malek et al. [22], show the protein composition of Amide I and Amide II regions including the ester group (1485–1760 cm^−1^) in hRPEs treated with H_2_O_2_, Rap, Baf, and Baf+H_2_O_2_.

In the protein region of the treated hRPEs (H_2_O_2_, Rap, Baf), the changes in the intensity and position of the Amide I band indicated conformational alterations in the secondary structure of the proteins compared to the control untreated cells. In all the treated samples, a dominant band centered around 1655 cm^−1^ was observed, which indicates the main protein conformation was α-helix. The average spectra of the treated samples showed absorbance intensity in the Amide I band (1656 cm^−1^) when compared to the control: it was lower in the H_2_O_2_ and Rap treated groups, and higher in the Baf and Baf+H_2_O_2_ treated groups as indicated by the blue arrows. The shift in the Amide I position can be indicative of conformational changes in the secondary structure of the proteins under such treatments, as observed in the Rap and Baf treated groups when compared to the control (Figure 1). This was confirmed after the second derivate of spectra in Figure S1. Treatment with H_2_O_2_ and Baf+H_2_O_2_ showed a sub-peak at ~1645 cm^−1^ which indicates more random coil structure in total proteins (Figure S1A,D). Baf treatment with and without the presence of H_2_O_2_ showed decreased absorption in the free amino acids chains (~1615 cm^−1^; orange arrow Figure 1). Besides, a shift towards lower wavenumbers and intensity decrease in the Amide II band (1540 cm^−1^) was also observed in the H_2_O_2_ and Rap-treated samples, showing the highest protein conformational changes during the treatment (Figure 1). An increase in the Baf+H_2_O_2_ treated cell group included a shift towards higher wavenumbers in this group as indicated by the red arrow. Interestingly, the Tyrosine band at ~1517 cm^−1^ was pronounced after H_2_O_2_ and Baf+H_2_O_2_ treatments (yellow arrow Figure 1 and Figure S1).

The band at ∼1740 cm^−1^ (green arrow) is assigned to the ester or carbonyl groups which are associated with the short lipids and carbonylated proteins content—types of protein or lipid oxidation modifications that can be induced by the presence of ROS, as well as markers for oxidative stress [23]. The absorbance intensity at ∼1740 cm^−1^ was the lowest in the Baf+H_2_O_2_ treated group, while the Rap treatment showed a more pronounced carboxyl group presence in the hRPEs around ~1720 cm^−1^. Also, an important effect of H_2_O_2_ and baf+H_2_O_2_ was a decrease in carboxyl groups at ~1719 cm^−1^ (Figure S1).

**Figure 2:**
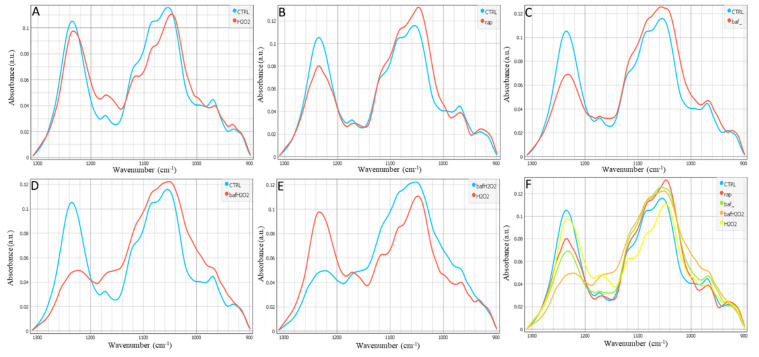
Average spectra of the fingerprint (nucleic acids and carbohydrates) region (890–1310 cm^−1^) in hRPE cells: (**A**) Control vs. H_2_O_2_, (**B**) Control vs. Rap, (**C**) Control vs. Baf, (**D**) Control vs. Baf+H_2_O_2_, (**E**) Baf+H_2_O_2_ vs. H_2_O_2_, and (**F**) all conditions are being shown. Representative data of the number of cells per condition (N = 35–37 cells).

Figure 2 and Table S1 display the area of the nucleic acids in the FTIR spectral range.

In the fingerprint region, two spectral areas appeared to be modified upon different treatments. There was a decrease in the intensity of the peak of asymmetric stretching of PO_4_^-^ groups at 1235 cm^−1^ in all treated groups (H_2_O_2_, Rap, Baf), which is associated with degradation of DNA; meanwhile, increased intensity of the peak at 1054 cm^−1^ associated to the presence of carbohydrates, was found when hRPEs were treated by Rap, Baf, and Baf+H_2_O_2_. The band around 970 cm^−1^ is associated with the phosphorylated proteins, and these were decreased after H_2_O_2_ and Rap treatments. The multiple changes in the absorbance and position (shift) at ~1086 cm^−1^ of the B form of DNA were pronounced in all the treatments in comparison to the control. These peaks indicate a number of different modifications of the DNA conformational changes or rearrangements attributed to the distortions of the DNA double helix in the presence of H_2_O_2_, Rap, or Baf.

The bands between 2800 and 3050 cm^−1^ mainly represent C–H stretching vibrations that are caused by lipids. This region of the spectrum consists of CH_2_ symmetric and asymmetric stretching represented by the intensities of the bands around 2852 cm^−1^ and 2922 cm^−1^, and CH_3_ asymmetric and symmetric stretching bands at 2956 cm^−1^ and 2870 cm^−1^, respectively [24].

**Figure 3:**
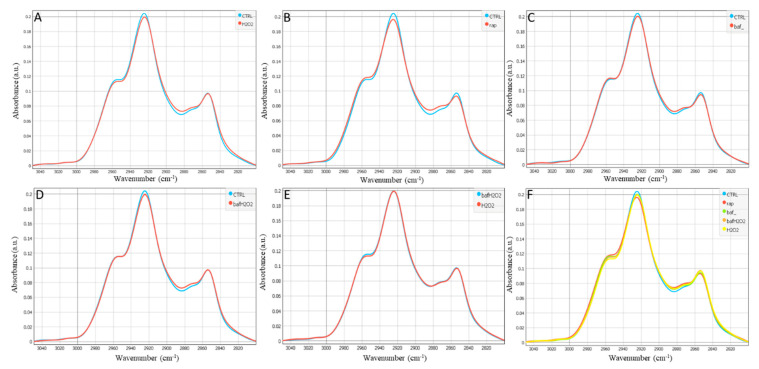
Average spectra of the lipid region (2800–3050 cm^−1^) in hRPEs: (**A**) Control vs. H_2_O_2_, (**B**) Control vs. Rap, (**C**) Control vs. Baf, (**D**) Control vs. Baf+H_2_O_2_, (**E**) Baf+H_2_O_2_ vs. H_2_O_2_, and (**F**) all conditions are being shown. Representative data of a number of cells per condition (N = 35–37 cells).

Figure 3 and Table S1 show that the different treatments (H_2_O_2_, Rap, Baf) can induce several modifications in the lipid spectral region, which may involve a wide range of biological processes. H_2_O_2_ treatment decreased the intensity of both asymmetric CH_3_ and CH_2_ bands at 2956 and 2924 cm^−1^, respectively, while only the CH_2_ asymmetric band was decreased by the Rap and Baf treatments compared to the control untreated cells. The vibrational asymmetric stretching of CH_2_ at 2924 cm^−1^ decreased in all the treatments compared to the control untreated cells, which can be related to the presence of any lipid disorders. Additionally, with the symmetric CH_2_ band at 2853 cm^−1^, a decrease could be observed in the Rap- and Baf- treated hRPEs accordingly.

In Figure 1, Figure 2 and Figure 3 there is no scattering of the infrared light over the cells as shown in Figure S2 due to the morphology of the hRPEs which display a plane rather than a round cell shape.

**Figure 4:**
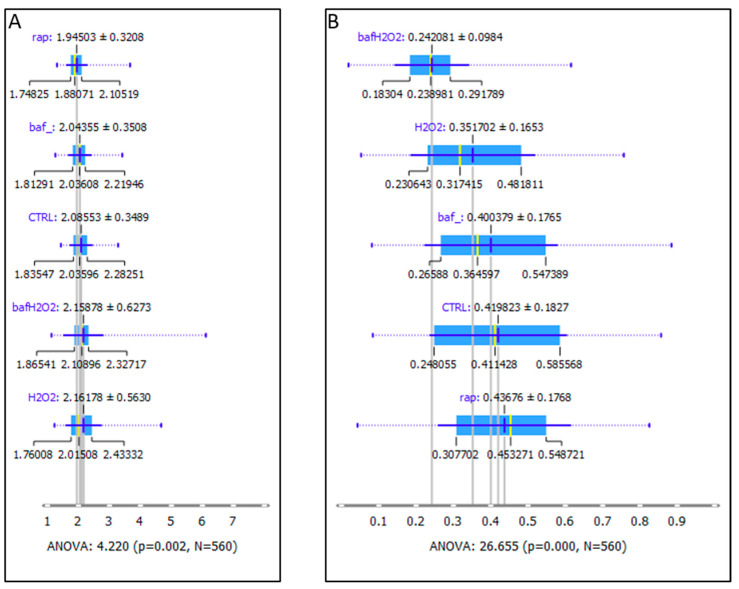
Analysis of (**A**) oxidative stress markers—the ratio of CH_2_ and CH_3_ asymmetric vibration bands are shown, as well as (**B**) the presence of C=O groups. Values are presented as mean ± SD.

Figure 4A presents the ratio of asymmetric vibrations of CH_2_ and CH_3_ bands_,_ which can be used as a potential marker for oxidative stress [25]. A significant difference, indicated by the p value under the image, can be seen between the control untreated cells compared to the H_2_O_2_ and other treated groups. H_2_O_2_-treated cells showed a higher value for lipid peroxidation, a process generated by the effect of several ROS, including H_2_O_2_. Figure 4B shows the integrated area of the carbonyl groups with the correlation between autophagy and apoptosis, but not oxidative stress.

**Figure 5:**
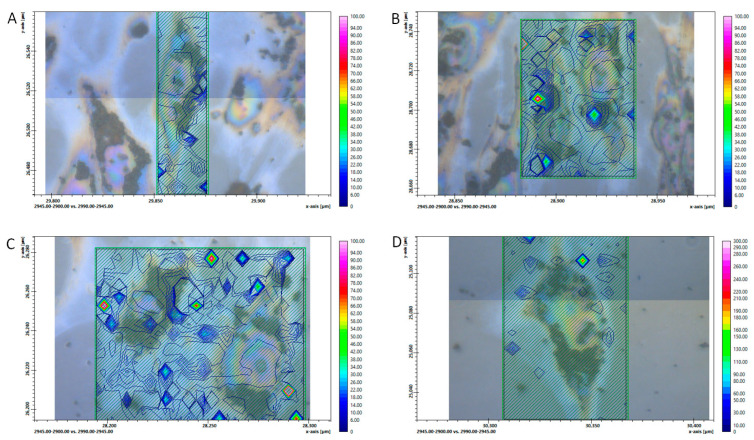
The ratio of CH_2_ and CH_3_ asymmetric vibration in a single hRPE cell mapping (**A**) Control, (**B**) H_2_O_2_- (**C**) Rap-, and (**D**) Baf+H_2_O_2_ treated cells. The color-coded images of the regions of interest representing the ratio are superimposed on the visible light bright field micrographies (field of view). In the cell cytoplasm, pigmentation in the form of melanin pigment granules typical for the primary hRPEs is visible around the clear nucleus.

**Figure 6:**
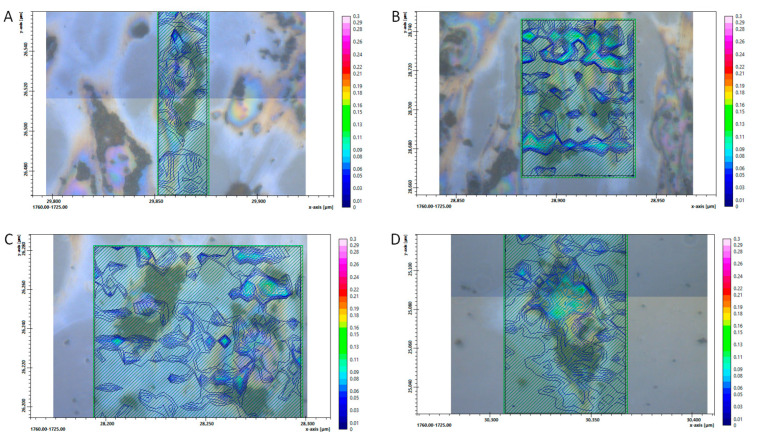
C=O in a single hRPE cell mapping: (**A**) Control, (**B**) H_2_O_2_- (**C**), Rap-, and (**D**) Baf+H_2_O_2_ treated cells. The color-coded images of the regions of interest representing C=O are superimposed on the visible light bright field micrographies (field of view). In the cell cytoplasm, pigmentation in the form of melanin pigment granules typical for the primary hRPEs is visible around the clear nucleus.

Besides the ratio of CH_2_ vs. CH_3_, the carbonyl group in relation to the vibration of CH_2_ and CH_3_ is also a good indicator of oxidative stress [26]. Figure 5 and Figure 6 show the FTIR images’ spatial distribution of the ratio of CH_2_ and CH_3_ asymmetric vibrations and the C=O allocation, respectively, in a single hRPE cell mapping for different treatment conditions. In Figure 5, the control cells show low oxidative stress on the periphery and within the cell cytoplasm, while H_2_O_2_- and Rap-treated cells showed a high density of the “hot spots” peripherally; the Baf+H_2_O_2_ appeared to show only a few isolated “hot spots” higher than the control cells. Figure 6 shows a stronger presence of carbonyl groups in H_2_O_2_ treated cells than Baf+H_2_O_2_ and Rap in comparison to the control untreated cells.

## 4. Discussion

Pathological changes in AMD are initiated in the RPE cells. This monolayer of cells is highly active throughout life, and is responsible for maintaining metabolic homeostasis and a stable microenvironment supporting the neurosensory retina. The key task of the RPEs is enabling the normal functioning of photoreceptors, which is achieved, among other mechanisms, by continuous phagocytosis of shed POS. The decrease in the number of RPEs over the years, and the constant exposure to oxidative stress due to high metabolic activities, results in an insufficient state of the RPE layer, incomplete POS digestion, and accumulation of waste material such as lipofuscin granules [27].

Many disease processes, particularly in age-related ones, have been linked to oxidative stress, which refers to cellular damage caused by reactive oxygen intermediates. The retina is one of the highest oxygen-consuming tissues in the human body and resides in an environment that is primed for the generation of ROS. Because of its lifetime light exposure, high energy demand, oxygen consumption, and amount of polyunsaturated fatty acids as well as high oxygen partial pressure from the underlying choriocapillaris, the retina, and its RPEs are exposed to chronic oxidative stress. With aging, oxidative damage increases, and the antioxidant capacity decreases as well as the repair system effectiveness. ROS can damage carbohydrates, membrane lipids, proteins, and nucleic acids, and this damage is thought to play a role in the development of many diseases [28,29].

When applied to eye cell research, SR-FTIR microspectroscopy can be a useful tool for the evaluation of oxidative stress in cells in situ. The technique has been used to study the oxidative damage in the retina in mice, where the ratios of several infrared (IR) bands, including an increase in nucleic acid/protein, a decrease in olefinic/lipid, and an increase in hydrocarbon acyl chains in lipids in a diabetic retina model have been reported [30]. Besides, the SR-FTIR has been used to investigate the UV effect on human lenses where oxidative stress markers have been shown to significantly increase lipid peroxidation with the diminishment of the asymmetric CH_3_ band after UV irradiation [31]. The same study showed that protein aggregation in the form of fibrils was prominent in the lens epithelial cells (LECs) of nuclear cataracts, while oxidative stress and lipids peroxidation were more pronounced in LECs of cortical cataracts [32]. Moreover, stress caused by ultrasound was shown to significantly decrease the soluble protein content of the cornea, while the use of antioxidants could reduce the adverse effects of ultrasound, upon the FTIR analysis of corneal tissue in New Zealand albino rabbits [33]. FTIR microspectroscopy enabled the evaluation of chemical changes in the photoreceptor outer segments exposed to ferrous sulfate, which promotes oxidative tissue damage [34].

In our study, hRPEs showed lower intensity in the Amide I band for the H_2_O_2_ and Rap treated (oxidative stress/autophagy induced) group, and higher in the Baf and Baf+H_2_O_2_ treated groups when compared to untreated samples indicating conformational alterations in the secondary structure of the cellular proteins. The H_2_O_2_-treated sample indicated a higher Tyrosine amino acid presence. The carbonyl group was also evaluated, and for all the treatments except Rap, it was significantly decreased in comparison to the control untreated cells.

The fingerprint profile in the region of 890–1310 cm^−1^ showed differential patterns for all treatment groups, associated with conformational changes in the DNA due to distortion of the double helix upon oxidative stress, and in the presence of autophagy inhibitor. The downward shift in the *ν*_as_PO_2_^−^ (named phosphate I) band at 1235 cm^−1^ suggests a conformational change in the DNA structure, and due to the nucleic acid phosphodiester groups modifications and nucleotide base damage, a partial shift between B-DNA and A-DNA has occurred [35,36]. Indeed, a reduction in the absorbance- of the phosphate I band has also been reported upon oxidative stress before [37].

The lipid spectral region was also affected by lipid peroxidation during induction and inhibition of autophagy. Such oxidative damage can affect the cellular membranes, vesicles, lipoproteins, and other molecules that contain lipids. Lipid peroxidation—an oxidative deterioration, where oxidants such as reactive oxygen species act upon lipids, has been implicated in the development of degenerative ocular disorders in the eye (AMD, cataract, glaucoma, diabetic retinopathy) [38,39]. In our study, the distribution of the ratios between the asymmetric CH_2_ and CH_3_ lipid bands estimated by following the lipids peroxidation can be used as a marker for oxidative stress [25,26,32]: a significantly higher value in the H_2_O_2_ treated group could be measured compared to the control group as a consequence of lipid peroxidation. Using different therapies that can target the imbalance in the redox system, one may restore the protein carbonylation and/or lipid peroxidation, and possibly avoid disease progression [40]. Lipid metabolism which is the synthesis and degradation of lipids to generate energy or produce the structural components of cell membranes interplays with autophagy as well.

Although there are other oxidative stress pathways linked to AMD, autophagy/heterophagy, and apoptosis are the most prominent ones [41]. The accumulation of auto-oxidative lipofuscin in the lysosomes of RPE cells, as well as drusen development in the extracellular space between the RPEs and the Bruch’s membrane, occurs when autophagy and/or heterophagy are affected, while this tendency worsens with age [7].

Autophagy is involved in processes that are critical for cell survival, such as the removal of altered cellular organelles, the elimination of viruses and bacteria from cells, and the prevention of altered protein accumulation [42,43]. In addition to its physiological function in the human eye, autophagy plays an important role in cellular homeostasis. Many of the cells in the retina have a high rate of cellular metabolism and a low rate of cell division or are highly differentiated [44]. Exposure to visible light and UV radiation exposes the cells in the eye to a highly oxidative environment, which can cause cellular damage [7]. Most cell types in the eye use autophagy as a cytoprotective mechanism to deal with oxidative damage [45,46]. In our study, the Rap treatment introduced the highest differences in all bio-macromolecular profiles, which can be used as possible future markers for AMD development and progression. Markers of autophagy have been discovered in the drusen of AMD donor tissue [47]. When RPE cells have been continuously exposed to oxidative stress caused by H_2_O_2_, the autophagy levels have decreased, while RPEs exposed to acute oxidative stress have experienced a higher presence of autophagy biomarkers.

Autophagy is normally engaged in early AMD due to compensatory mechanisms that increase after oxidative stress in the RPEs. However, in late AMD, the autophagy pathway is unable to combat a large number of damaged organelles and hence becomes dysfunctional [48]. Failure of RPEs to utilize autophagy as a survival mechanism can lead to a buildup of aggregation-prone proteins, cellular degeneration, and eventually cell death. Constant oxidative stress affects autophagy and heterophagy, promotes protein aggregation, and activates the inflammasome, all of which contribute to the pathogenic phenotype of AMD [5].

The presence of oxidative damage in the retina after autophagy inhibition suggests that autophagy may serve as an important antioxidant system in the retina. Furthermore, autophagy dysfunction has been demonstrated to cause inflammation by attracting inflammasome-activated macrophages [49].

To the best of our knowledge, SR-FTIR has not been used before for studying autophagy in the eye’s primary cell culture. In human colorectal adenocarcinoma HT-29 cancer cells, FTIR has been used to study the differences in the shape of the Amide I and II bands in the region of proteins under starvation-induced autophagy; relevant differences in these band regions under normal and starvation conditions could be shown, as well as relevant changes in the spectral signature at approximately 1595 cm^−1^ as potential biomarkers of starvation-induced autophagy [50].

In a murine model of multiple sclerosis, there was a link between protein carbonylation (but not oxidative stress) and apoptosis, with higher carbonyl levels in apoptotic cells than in live cells, and decreased autophagy in both acute and chronic experimental autoimmune encephalomyelitis [51]. Furthermore, it was shown that Baf suppresses and kills leukemic primary cells, inhibits and attenuates cytoprotective autophagy, promotes apoptosis, and delays the development of leukemia in a xenograft mouse model [52]. The treatment by Baf could in our study somehow “accurate” spectra of the bio-macromolecules in comparison to the Rap-induced ones (Figure 1B,C, Figure 2B,C and Figure 3B,C).

In our autophagy and oxidative stress ex vivo AMD study model, we observed a higher presence of C=O groups probably due to protein carbonylation in the Rap-induced autophagy group compared to the control untreated cells. Moreover, while Baf targets both autophagy and apoptosis in a way that inhibits autophagy and induces apoptosis simultaneously, H_2_O_2_ has also been used as an inducer of apoptosis. As a consequence of that, we could observe protein carbonylation in the Baf and H_2_O_2_ treated cells also. Likely, the apoptosis-associated membrane changes in the Baf, H_2_O_2_, and Baf+H_2_O_2_ treated cells could explain the presence of the C=O group.

## 5. Conclusions

The role of autophagy and oxidative stress in AMD is a much-studied phenomenon. Autophagy is altered during AMD, with induction and inhibition models for this cellular degradation mechanism in hRPEs being much needed. This study shows that oxidative stress/autophagy can induce changes in hRPEs detected by highly-sophisticated non-destructive SR-FTIR microspectroscopy and imaging analysis; the findings demonstrate the cellular fingerprint of the biomacromolecular changes of hRPEs in an ex vivo model for AMD. The changes in the phosphate groups of nucleic acids, Amide I and II of the proteins, the carbonyl groups, and the lipid status in the hRPEs showed a significantly different pattern under oxidative stress/autophagy induction and inhibition. This biomolecular fingerprint can be evaluated in the future when discovering new drug targets which can affect autophagy and oxidative stress in AMD.

## Data Availability

All data related to this work can be made available upon request to the corresponding authors.

## Supplementary Materials

The following supporting information can be downloaded at: https://www.mdpi.com/article/10.3390/biomedicines11020300/s1, Figure S1: The second derivative of the average spectra of the protein and ester regions (1485–1760 cm^−1^) in hRPE cells: (A) Control (untreated) vs. H_2_O_2_, (B) Control vs. Rap, (C) Control vs. Baf, (D) Control vs. Baf+H_2_O_2_, (E) Baf+H_2_O_2_ vs. H_2_O_2_, and (F) all conditions are being shown. Representative data of number of cells per condition (N = 35–37 cells); Figure S2: FTIR spectra of the cells, including the finger print (nucleic acids and carbohydrates), protein and ester regions, and lipid region in hRPEs: blue (Control), red (Rap), green (Baf), orange (Baf+H_2_O_2_) and yellow (H_2_O_2_). The band at 2350 cm^−1^ annotated carbon dioxide which is not relevant for our data. Representative data of the number of cells per condition (N = 35–37 cells) are being shown; Table S1: Assignment of FTIR bands and their changes under different treatments of hRPEs with a colour-coded representation of the absorbance intensity (white-unchanged, green-increased and red-decreased).
