# Supplementary material for: Biomacromolecular Profile in Human Primary Retinal Pigment Epithelial Cells—A Study of Oxidative Stress and Autophagy by Synchrotron-Based FTIR Microspectroscopy
Source: Biomedicines. 2023 Jan 21;11(2):300. doi: 10.3390/biomedicines11020300 (PMC9952973; doi:10.3390/biomedicines11020300)
Supplement: Supplementary file 1 [file biomedicines-11-00300-s001.zip › biomedicines-2142326-supplementary.pdf]

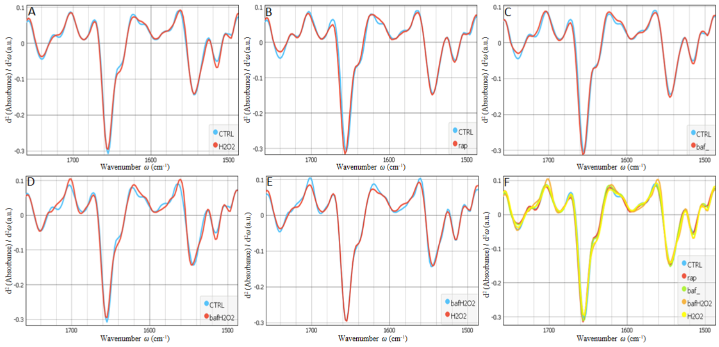

Figure S1: The second derivative of the average spectra of the protein and ester regions (1485–1760 cm^{-1}) in hRPE cells: (A) Control (untreated) vs. H_2O_2, (B) Control vs. Rap, (C) Control vs. Baf, (D) Control vs. Baf+H_2O_2, (E) Baf+H_2O_2 vs. H_2O_2, and (F) all conditions are being shown. Representative data of number of cells per condition (N = 35–37 cells).

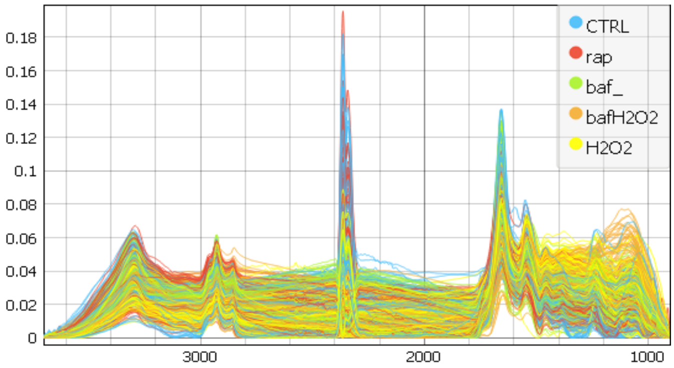

Figure S2: FTIR spectra of the cells, including the finger print (nucleic acids and carbohydrates), protein and ester regions, and lipid region in hRPEs: blue (Control), red (Rap), green (Baf), orange (Baf+H_2O_2) and yellow (H_2O_2). The band at 2350 cm^{-1} annotated carbon dioxide which is not relevant for our data. Representative data of the number of cells per condition (N = 35–37 cells) are being shown.

				Treatment of hRPEs			
Vibrational mode	Wavenumber (cm^{-1})	Assignment	control	H_2O_2	Rap	Baf	Baf+H_2O_2
CH_3 asymmetric stretch	2956	Lipids and proteins					
CH_2 asymmetric stretch	2924	Saturated lipids and side chains of proteins					
CH_2 symmetric stretch	2853	Saturated lipids and side chains of proteins					
C= O stretch (ester)	1720-1740	Lipids, phospholipids					
C= O stretch+NH bend (Amide I)	1656	Proteins—α-helix structure					
Ring CC stretch of Tyr and Phe residues, and nucleotides	1615	Proteins, nucleic acids					
NH bend+C-N stretch (Amide II)	1540	Proteins					
Ring CC stretch of Tyr residues	1517	Tyrosine proteins					
PO_2^- asymmetric stretch	1235	DNA					
PO_2^- symmetric stretch	1089	Nucleic acids, phospholipids					
COH deformation	1050-1056	Mucin, carbohydrates					
Dianionic phosphate monoester	970	Phosphorylated proteins					

Table S1: Assignment of FTIR bands and their changes under different treatments of hRPEs with a colour-coded representation of the absorbance intensity (white-unchanged, green-increased and red-decreased).
